# Editorial: Risk of Dietary Hazardous Substances and Impact on Human Microbiota: Possible Role in Several Dysbiosis Phenotypes

**DOI:** 10.3389/fmicb.2021.669480

**Published:** 2021-04-23

**Authors:** Margarita Aguilera, Bruno Lamas, Els Van Pamel, Mangesh Bhide, Eric Houdeau, Ana Rivas

**Affiliations:** ^1^Department of Microbiology, Faculty of Pharmacy, University of Granada, Granada, Spain; ^2^Department of Microbiology, Instituto de Investigación Biosanitaria ibs, Granada, Spain; ^3^Research Center in Food Toxicology (Toxalim), INRAE, Toulouse University, ENVT, INP-Purpan, UPS, Toulouse, France; ^4^Flanders Research Institute for Agriculture, Fisheries and Food (ILVO), Merelbeke, Belgium; ^5^Department of Microbiology and Immunology, Institute of Immunology, University of Veterinary Medicine and Pharmacy, Kosice, Slovakia

**Keywords:** xenobiotics, endocrine disruptors, microbiota, dysbiosis, probiotics

The cumulative dietary exposure to hazardous substances such as endocrine disruptor chemicals (EDC) that are present in common and processed food is continuously increasing (Gálvez-Ontiveros et al., 2021). Prolonged exposure to EDC seems to affect human health by triggering obesity, insulin resistance, metabolic syndrome, and even infertility (Baker et al., 2017). Unfortunately, there is still a lack of knowledge on specific biomarkers necessary to better understand the mechanisms associated to specific EDC-related pathogenesis. The impact of EDC has been traditionally investigated through biomonitoring and epidemiological studies. However, analytical determinations of EDC in biological samples failed to provide consistent results. Thus, a paradigm shift in EDC-related disorders should take into consideration new scientific evidence supporting the fact that EDC may influence human health by modifying the human microbiota, as well as compiling evidence on the existence of microorganism species in the gut that are able to biodegrade such obesogens or contaminants (Gálvez-Ontiveros et al., 2020; López-Moreno et al., 2021). In this sense, relevant studies also support the development of predictive toxicological models that account for the gut-microbiota-immune axis (Lamas et al., 2020). For example, gut dysbiosis and immune system dysfunctions precede obese phenotype development in mice perinatally exposed to bisphenol A (BPA), a common EDC in human food (Malaisé et al., 2017, 2018). Therefore, discovering the association and role of microbiota as a metabolic mediator of food xenobiotics and endocrine pathophysiological outcomes has resulted in building multidisciplinary and integrative hypotheses. This research also provides opportunities to use microbial modulators like probiotics for restoring the dysbiotic taxa in EDC-related disorders (López-Moreno and Aguilera, 2020; López-Moreno et al., 2020). Importantly, EDC food risk assessment in relation to human microbiome variability and health outcomes is still at its very first phase of development, requiring integrative expertise and holistic analyses, i.e., a systemic approach.

The current Research Topic covers a collection of reviews, mini reviews, and original research articles focusing on dietary xenobiotics, human microbiota dysbiosis, and probiotics or prebiotics for modulating the associated pathophysiological phenotypes. In this sense, reviews agreed on the association of health disorders due to the impact of xenobiotics on the occurrence of microbiota dysbiosis.

In their mini review, Barnett and Gibson covered glyphosate's effects on the gut microbiome diversity and concluded that glyphosate residues on food could cause dysbiosis, given that opportunistic pathogens are more resistant to glyphosate compared to commensal bacteria. Moreover, glyphosate could inhibit exclusive pathways of bacteria, like the shikimate pathway. In addition, they postulated that the wheat sensitivity induced by the Western diet rich in high carbohydrates, seemed to be not only due to gluten but also to herbicides used to promote higher crop yields, and as a result of increased industrialized and processed food needs.

In their review, Aguilera et al. extensively analyzed the interactions of steroid hormones and EDC with gut microbiota. The authors innovatively suggest the term “endobolome” for stating gut microbiota genes and pathways involved in EDC metabolization. Microbiota disrupting chemicals (MDC) is the term to group the dietary xenobiotics that alter gut microbial components linked to chronic intestinal and systemic diseases. MDC allow distinguishing the role of contaminants from other microbiota natural modifiers. It is possible to develop specific research on the triad MDC-microbiota-host health axis.

Several original research papers published in this Research Topic focus on gut dysbiosis and pathogens, specifically on the characterisation of molecular pathways involved in the gut dysbiosis and possible modulation strategies. In this sense, the findings reported by Rubio-Gómez et al. suggest that dysbiosis or depletion of gut microbiota by antibiotics facilitated the colonization of the intestinal tract by multidrug-resistant pathogens (Pseudomonas aeruginosa PAO1). Fructooligosaccharides (FOS) and bifidogenic effects selectively reduced bacterial pathogenicity, decreasing growth, motility and biofilm by interfering with different canonical and signaling pathways. Wang et al. focused their study on *Vibrio parahaemolyticus*, which is a food-borne pathogen that can cause gut dysbiosis, disorders, diarrhea, and abdominal pain depending on the colonizer strain. Specific genes (*tdh* and *trh*) were only present in *V. parahaemolyticus* pathogenic strains, which showed strong activity to simulated gastric fluids. The study provided an innovative understanding to unlock the efficient control of pathogenic *V. parahaemolyticus*.

Three other research articles using mice models with induced gut dysbiosis focused on discovering mechanistic aspects after exposure to xenobiotics and obesogens. Li et al. showed that food additives such as polysorbate 80 (P80) exacerbate irradiation-induced gastrointestinal (GI) tract toxicity, microbiota dysbiosis, and tract injury. In summary, their findings demonstrated that P80 is a potential risk factor for cancer patients during radiotherapy and indicated that microbial butyrate might be a therapy to mitigate the co-morbidities.

Lin et al. tested, in high-fat diet (HFD)-induced obese mice, the effects of *Lactobacillus kefirnofaciens* M1 and *Lactobacillus mali* APS1, which are probiotics with, respectively, obesogenic and anti-obesogenic properties. The authors observed that M1/APS1 intervention influenced fat accumulation by regulating adipogenesis and inflammation-related marker expressions. Moreover, particular gut microbiota family taxa (*Christensenellaceae* and *Bacteroidales* S24-7) were negatively correlated with body weight gain through an increase in the esterified carnitine (acylcarnitine), which is essential for lipid intermediary metabolism and for energy expenditure and storage balance.

Liu et al. proved that the heavy metals arsenic (As), cadmium (Cd), and lead (Pb) modify the risk of disease by mediating on gut microbiota dysbiosis. Disease risk of As, Pb, and Cd exposure was more severe in HFD-fed mice compared to normal diet (ND)-fed mice, possibly due to a higher accumulation of heavy metals in the liver and kidneys. Specific increased microbes seemed to play a role in the heavy metal detoxification *via* absorption and faecal excretion. In ND-fed mice, relative abundance of *Bacteroides, Lactobacillus, Butyricimonas*, and *Dorea* increased after As exposure; *Faecoccus* and *Lactobacillus* increased after Cd exposure; and *Desulfovibrio*, and *Roseburia* increased after Pb exposure, while no difference in these same taxa was observed in HFD-fed mice microbiota. In summary, the enriched microbes that might respond to heavy metals in ND-fed mice did not respond or could not be enriched accordingly in HFD-fed mice.

Ren et al. described evidence that the natural dietary flavonoid, acacetin, attenuates dextran sulphate sodium (DSS)-induced colitis in mice by inhibiting inflammation (macrophages and inflammatory mediators). Acacetin was also able to restore the altered gut diversity microbiota profile caused by DSS reversing the reduction of Firmicutes, and the enrichment of Proteobacteria, Bacteroidetes, and Deferribacteres phyla. Overall, the results suggest that acacetin alleviated weight loss, diarrhea, colon shortening, inflammatory infiltration, and histological injuries.

Finally, xenobiotics in a diet and the environment could also affect other microbiotas encountered at body locations different from the gut. In this direction, Gonzalez et al. discovered temporal changes to the breast milk microbiome of healthy Guatemalan mothers from early to late lactation, according to several maternal factors and exposures. These included a general shift from *Staphylococcus* and *Streptococcus* species in early lactation to Sphingobium and Pseudomonas species in late lactation. Interestingly, species enriched in early lactation included putative commensal bacteria known to colonize the infant oral and intestinal tracts, whereas species enriched in late lactation, such as *Sphingomonas yanoikuyae, Pseudomonas putida, Stenotrophomonas maltophilia, Cloacibacterium normanense, Comamonas testosteroni, Ottowia beijingensis*, and *Flavobacterium cucumis*, showed common functional traits associated with hazardous substance biodegradation, like steroids and more general polycyclic aromatic hydrocarbon pathways.

In summary, together the articles in this Research Topic make a significant contribution to the evoked dysbiosis of the microbiome following exposure to EDC, as a step toward a better comprehension of the endocrine-related pathophysiology.

## Author Contributions

MA: conceptualization and study design. AR: validation. MA, BL, EVP, EH: writing—original draft preparation. All authors: revision. All authors listed have made a substantial, direct and intellectual contribution to the work, and approved it for publication.

## Conflict of Interest

The authors declare that the research was conducted in the absence of any commercial or financial relationships that could be construed as a potential conflict of interest.
